# Future healthcare providers and professionalism on social media: a cross-sectional study

**DOI:** 10.1186/s12910-022-00742-7

**Published:** 2022-01-20

**Authors:** Rabih Soubra, Ibrahim Hasan, Louna Ftouni, Adam Saab, Issam Shaarani

**Affiliations:** Faculty of Medicine, Beirut Arab University, Riad El Solh, 11-5020, Beirut, 11072809 Lebanon

**Keywords:** Education, Ethics, Healthcare students, Professionalism, Professional conduct, Social media

## Abstract

**Background:**

Nowadays, social media have become central in the daily lives of people, including healthcare professionals. Fears arise that the accelerated growth of these social platforms was not accompanied by the appropriate training of the healthcare students and workers on the professional use of social media. This study primarily aimed to assess the awareness of the healthcare students at Beirut Arab University, Lebanon on the professional standards of social media. It also aimed to assess the presence of differences in the practices and attitudes of healthcare students according to gender and major.

**Methods:**

A cross-sectional study was designed, and a paper-based questionnaire was distributed to healthcare students. Chi-Square test was used to analyse certain findings.

**Results:**

Out of 1800 students approached, 496 participated in the questionnaire. All participants used social media. Only 19.5% (96/496) of them had received a structured education on the professional use of social media during their university study. The majority of students (349/488, 71.5%) thought that the professional standards on social media are distinct from those established in face-to-face interactions. Female students were more likely to get adequate answers in accordance with international guidelines. There were statistically significant differences in the practices and attitudes of students belonging to different majors (*p* value < 0.05).

**Conclusion:**

The line between what is professional on social media, and what is not, remains blurred for healthcare students. This study uncovered the need for clear and updated evidence-based guidelines assisting students in making the most appropriate decisions in the various online scenarios faced in healthcare practice.

## Background

Since the ancient times, Medicine and Ethics emerged inseparable in the approach to heal the sick. Throughout history, thousands of texts attempted to dictate the framework of medical ethics, the cornerstone of which, was the Hippocratic oath. To date [1, 2], this oath traces a scholar discipline of moral principles to modern medicine thus striking it as the most humane of sciences. However, Hippocrates and all who came after did not predict that centuries from then, a global phenomenon would take the world by storm and force a change in the principles of the clinical-moral code [3]*.* This phenomenon is the emergence of social media. Social media can be defined as “forms of media that allow people to communicate and share information using the Internet” [4]. These virtual modern ways of communication made online presence a societal norm, changing the face of human–human interactions, and urging healthcare professionals to conform to these changes [5]. Social media offer many benefits to healthcare professionals. These benefits include, but are not limited to, the creation of highly trusted professional pages that provide patients with evidence-based and updated information about their conditions, the establishment of professional rapport between international healthcare providers for the exchange of novel techniques for patient care, and the promotion of research matters [3, 6–9]. However, ambiguity prevails in the healthcare sector over the moral principles on social media, and modern-day healthcare professionals are finding themselves facing daily emerging ethical dilemmas [10, 11]*.* Consequently, a form of vagueness has risen in adopting a professional approach on the various social media platforms [12].

Studies dating from the past decade showed that doctors have limited presence on social media and are unsure as to how to respond to modern ethical dilemmas [10, 13]. One of the most relevant of these issues is privacy. As one study has pointed out [11], the undefined borders between private life and professional life are compromising the patient-healthcare professional relationship due to private information gains on either side [14].

One of the factors that threaten professionalism worldwide is the unpreparedness of healthcare providers to transition to virtual interactions with patients [15–17], hence the need to promote professionalism in medical education has risen and is constantly being modified [18–20]. Some studies on healthcare students [21, 22], the majority of which done in Western and Westernized countries, showed the importance of implementing guidelines on the professional use of social media, but a limited number of studies [23–25] targeted all categories of healthcare students. In Lebanon, we found no research to date tackling social media use and professionalism, neither on students nor on healthcare workers. This study, to the best of our knowledge, is the first of its kind in Lebanon. It primarily aimed to assess the awareness of the students from different healthcare faculties—Medicine, Pharmacy, Dentistry, and Health Sciences encompassing Nursing, Physiotherapy, Medical Lab, and Nutrition—at a single institution “Beirut Arab University” on the professional standards of social media. It also aimed to detect differences in the practices and attitudes of healthcare students according to gender and major. Our secondary objective was to compare the awareness of pre-clinical versus clinical students to the available guidelines.

### Definition of pre-clinical and clinical years according to each major at Beirut Arab University

The pre-clinical years are the years in which students gain most of the core theoretical knowledge regarding their field of study, without interacting with patients. In contrast, the clinical years are the ones spent mostly in direct contact with patients.

At the faculty of Medicine, the first three years are the pre-clinical ones, while the latter three are the clinical years. At the faculty of Pharmacy, students from the second to the fifth year interact with patients through training in pharmacies. At the faculty of Dentistry, the third to fifth years are the clinical ones. Lastly, at the faculty of Health Sciences, Nursing students start their clinical training from the first year, Physiotherapy students start from the second year, Medical Lab students from the third year, while Nutrition students have no access to clinical training until after their graduation.

## Methods

### Study design and setting

We conducted a descriptive cross-sectional study between August 2017 and December 2019.

In February 2018 and following the approval of the Institutional Review Board (IRB) of Beirut Arab University (BAU) *(approval number: 2017H-0056-M-R-0224)*, we approached students of the Healthcare faculties at BAU, in Beirut, Lebanon, with written consent forms and paper-based questionnaires. We distributed the questionnaires in lecture rooms, halls, and libraries. We then collected the papers either from the registrar of the faculties or directly after completion of the questionnaires. We ensured the anonymity of the participants by not including any identifier in the questionnaire.

### Sample size

We expected a proportion of 50% of healthcare students to be aware of guidelines on the professional usage of social media. Using a power of 80%, a confidence level of 95%, and a margin of error of 5%, we set the estimated sample size at 385 through the following formula X = Z_α/2_^2^ *p*(1−*p*)/MOE^2^ [26]. To account for incomplete questionnaires (i.e., up to 20% incomplete), we determined the minimum number of participants at 480.

### Participants

The study included participants from the faculties of Medicine, Dentistry, Pharmacy, and Health Sciences. The latter encompasses the departments of Nursing, Medical Lab, Physiotherapy, and Nutrition. We included students from all academic years in this study. We ensured the representation of both male and female students.

### Questionnaire

We developed the questionnaire after a thorough literature review [10–12, 21, 22, 27–31]. We then carefully reviewed the questions and decided on answers that represent more ethical consensus [11, 22, 27, 28]. Lastly, we revised and piloted the final draft of the questionnaire.

We divided the questionnaire into sections targeting the participants’ demographics, characteristics of social media use, practices, and attitudes to assess students’ awareness on the matters of professionalism in online interactions and presence. In the demographics section, we asked questions related to the participants’ age, gender, major, and academic year. In the characteristics of social media use section, we asked about the platforms used by participants, the number of hours spent on these platforms, and the participants’ knowledge about the current privacy settings of their accounts. In the practices section, questions were related to sending and accepting friend requests from patients, interacting online with patients, and providing them with medical advice on social media platforms. In the final section of attitudes, the questions assessed the presence of difference in healthcare standards applied online versus in face-to-face interactions, responding online to patients’ concerns about their medical conditions, interacting online with patients who accessed their personal information, and professional conduct online (e.g., breaching patient confidentiality, posting controversial photographs, …). We placed these questions in a "Yes or No" format (a few were in a "Yes/No/Neutral" format) and in tables containing rating-scale questions of the "Likert-type scale".

We compared the questions in the attitudes section to those in established guidelines (General Medical Council “GMC”, General Dental Council “GDC”, Nursing and Midwifery Council “NMC”, General Pharmaceutical Council “GPC” and Royal Pharmaceutical Society “RPS”, British Dietetic Association “BDA”, Chartered Society of Physiotherapy “CSP”, and Canadian Society for Medical Laboratory Science “CSMLS”) to identify whether students’ attitudes conform to set standards and can be deemed appropriate. We found a general agreement among all the guidelines on eight of our questions in the attitudes section.

### Statistical analysis

We manually entered the collected data on International Business Machines (IBM) Statistical Package for the Social Sciences (SPSS) version 25. We reported descriptive statistics as percentages and frequencies for categorical variables and as means and standard deviations for continuous variables. We used the Chi-Square test to study associations between categorical variables. We considered a *p* value of less than 0.05 to be statistically significant.

## Results

### Participants

We approached 1800 students from the healthcare faculties, of which 496 filled out the questionnaires, yielding a response rate of 27.54%. The age of participants ranged from 17 to 31. Most of the participants (343/496, 69.2%) were females. Each faculty was represented in approximately 25% of the participants, and the majority were from the first three academic years (Table 1).

**Table 1:** Demographics of participants (*N* = 496)

Age (years)	
Mean	20.7 (SD* = 1.879)
Range	17 to 31
	N (%)
Gender	
Male	153 (30.8)
Female	343 (69.2)
Faculty/major	
Medicine	124 (25.0)
Pharmacy	111 (22.4)
Dentistry	134 (27.0)
Health sciences	127 (25.5)
Nursing	29 (5.8)
Nutrition	30 (6.0)
Physiotherapy	39 (7.9)
Medical laboratory	29 (5.8)
Academic year	
First^†^	98 (19.8)
Second^†^	115 (23.2)
Third^†^	114 (23.0)
Fourth^‡^	91 (18.3)
Fifth^§^	64 (12.9)
Sixth**	14 (2.8)

*SD means standard deviation

^†^Includes all faculties and majors

^‡^Includes faculties of Medicine, Pharmacy, Dentistry, and the Physiotherapy major

^§^Includes faculties of Medicine, Pharmacy, and Dentistry

^**^Includes faculty of Medicine only

### Social media usage

More than half of the participants (279/493, 56.6%) spent more than 3 h on social media per day. Most reported using Instagram the most (419/496, 84.5%), followed by Facebook (405/496, 81.7%), while the least used platforms were blogging websites such as WordPress and Tumblr (13/496, 2.6%). The majority of the participants (413/493, 83.8%) reported knowing the current privacy settings of their social media accounts (Table 2). Also, 80.5% (396/492) did not receive any structured education about the professional use of social media during their university study.

**Table 2:** Participants’ characteristics of social media use (*N* = 496)

	N (%)
*Hours spent per day using Social Media platforms*^§^	
Up to 1 h	36 (7.3)
Up to 2 h	65 (13.2)
Up to 3 h	113 (22.9)
Up to 4 h	91 (18.5)
More than 4 h	188 (38.1)
*Social media platforms used*	
Instagram	419 (84.5)
Facebook	405 (81.7)
YouTube	372 (75.0)
Snapchat	336 (67.7)
Google+	161 (32.5)
Twitter	83 (16.7)
LinkedIn	33 (6.7)
Blogging websites	13 (2.6)
Privacy settings of social media accounts known to participant*	413 (83.8)

*Some participants did not respond to this question

### Practices of healthcare students in online interactions with patients

A small minority of participants (13/227, 5.7%) reported that they had sent a friend request to a patient (Table 3). However, half of them (118/227, 52.0%) had received friend requests from patients, of which 58.1% (72/124) accepted the request, 7.3% (9/124) simply declined, 5.6% (7/124) declined the request after explaining the reason to the patient, and 29.0% (36/124) left the friend request pending. More than half of the participants stated their willingness to interact with patients on social media (302/487, 62.0%) and even provide medical advice to them (301/486, 61.9%).

**Table 3:** Practices on social media by Healthcare students regarding online interaction with patients (N = 496)

Statements	Yes answer N (%)	N
Had sent a friend request to a patient*^†^	13 (5.7)	227
Had received a ‘friend request’ on a social media account from a patient*^†^	118 (52.0)	227
Had accepted the friend request from a patient on social media account*^†^	72 (58.1)	124
Would interact, in the future, with a patient via social media*	302 (62.0)	487
Would send, in the future, a friend request to a patient*	151 (30.8)	490
Would accept, in the future practice, a friend request from a patient on social media*	228 (59.1)	487
Would provide medical advice to a patient on social media*	301 (61.9)	486
Would include information of medical importance learned about a patient online as a part of his/her medical record*	176 (36.4)	484

*Some participants did not respond to this question

^†^The response rate was lower in these questions because some participants were not eligible to respond if they have not encountered patients yet or have not received a friend request from a patient

When comparing the practices between male and female students, we found an association between gender and receiving friend requests from patients (*p* value = 0.016), with female students being more prone to receiving such requests on social media (57.5%, 88/153) compared to their male counterparts (40.5%, 30/74). There are statistically significant differences in the practices of students from the various majors regarding some aspects of social media usage. Regarding sending friend requests to patients (*p* value = 0.022), the vast majority of pharmacy students (97.7%) refrained from sending any request, while 16.7% of physiotherapy students have already sent a request. Concerning receiving friend requests from patients (*p* value = 0.002), dentistry students were the most liable to such requests (74.2%), compared to only 35.7% of medical students. For responding to patient’s friend request (p-value = 0.015), nursing and medical lab students accepted the highest number of requests (100% for each), in contrast to just 40% of physiotherapy students. Finally, for willingness to provide medical advice to patients on social media (*p* value = 0.000), 78.2% of pharmacy students expressed their desire to provide such advices, while medical students were the least prompted to provide them (46.8%).

### Attitudes of healthcare students towards various issues related to social media professionalism

The majority of students (349/488, 71.5%) stated that the professional standards of healthcare providers through social media differ from those in traditional face-to-face interactions. More than half of the participants (305/483, 63.1%) felt obliged to respond to a patient’s concerns about his/her medical condition and/or treatment if a patient established interaction with them through social media. Only 25.7% (125/487) were comfortable interacting with a patient who has accessed their personal information on social media. However, more participants (168/489, 34.4%) stated that they would feel comfortable if the information accessed by the patient were those tagged by others and not uploaded by the participants themselves. Mentioning a patient’s real name in a post on social media was regarded as inappropriate by 74.5% (360/483) of the participants (Table 4), while only 51.2% (246/480) considered discussing anonymized patient matters in online forums as inappropriate.

**Table 4:** Attitudes of Healthcare students on online platforms regarding social media professionalism (N = 496)

Appropriateness for a healthcare provider to do the following on social media	Appropriate	Neutral	Inappropriate
	N (%)*		
Interact with a patient^†^	233 (47.9)	177 (36.4)	76 (15.6)
Establish an “online friendship” with a patient^†^	109 (22.5)	221 (45.6)	155 (32)
Look up information about a patient^†^	126 (26.1)	172 (35.6)	185 (38.3)
Upload a picture showing a patient during a procedure^†^	36 (7.5)	70 (14.5)	376 (78)
Discuss individual patient matters in online forums if the patients are de-identified^†^	90 (18.8)	144 (30)	246 (51.2)
Complain about the attitudes and behaviors of patients anonymously^†^	56 (11.6)	125 (25.9)	302 (62.5)
Discuss his/her political views^†^	61 (12.6)	111 (22.9)	312 (64.5)
Discuss religion^†^	61 (12.6)	93 (19.2)	331 (68.2)
Write about his/her own social problems^†^	58 (12)	98 (20.2)	328 (67.8)
Mention the patient's real name in a post^†‡^	31 (6.4)	92 (19)	**360 (74.5)**
Mention any patient’s information that might indirectly lead to the patient identity^†‡^	22 (4.6)	56 (11.6)	**405 (83.9)**
Complain about his/her profession^†‡^	38 (7.8)	99 (20.4)	**348 (71.8)**
Complain about attitudes and behavior of colleagues^†‡^	32 (6.6)	88 (18.2)	**364 (75.2)**
Complain about attitudes and behavior of faculty members^†‡^	42 (8.7)	80 (16.5)	**363 (74.8)**
Publish his/her photos in bathing suits^†‡^	43 (8.9)	92 (19)	**349 (72.1)**
Publish photo of his/herself consuming alcohol^†‡^	46 (9.5)	104 (21.4)	**336 (69.1)**
Publish photo of his/herself smoking^†‡^	50 (10.3)	142 (29.2)	**294** **(60.5)**

*Percentages may not add to 100% due to rounding of figures

^†^Some participants did not respond to this question

^‡^The correct answer to these questions is “inappropriate” according to General Medical Council (GMC), General Dental Council (GDC), Nursing & Midwifery Council (NMC), General Pharmaceutical Council (GPC), Royal Pharmaceutical Society (RPS), British Dietetic Association (BDA), Chartered Society of Physiotherapy (CSP), Canadian Society for Medical Laboratory Science (CSMLS)

We determined whether there were associations between the eight questions agreed on in the guidelines (Table 4) and students’ gender and major. We found statistically significant differences in the attitudes of female and male students in six of eight questions, with answers of female students being more in line with the guidelines’ statements. The two questions in which no statistical significance was found were related to mentioning a patient’s real name in a post (*p* value = 0.506) and mentioning patient information that might indirectly lead to their identification (*p* value = 0.112). According to students’ majors, the only statistical significance was found in three out of the eight questions agreed on in the guidelines (Table 4). First, for mentioning a patient’s real name in a post (*p* value = 0.009), physiotherapy students obtained the highest number of correct answers (87.2%), in contrast to only 48.1% of nursing students. Second, for complaining about attitudes and behaviors of faculty members (*p* value = 0.037), 81.8% of pharmacy students complied with the guidelines, while only 50% of nursing students answered correctly. Third, for posting a photo on social media while smoking (*p* value = 0.034), 82.1% of physiotherapy students conformed to the international guidelines compared to about only half of the dentistry students (54.3%).

### Analysis of the attitudes of pre-clinical versus clinical students taking into account the guidelines stand mentioned in Table 4

About half of the students (226/483, 46.8%) had no more than one wrong answer in the specified attitudes questions which are in agreement with international guidelines, with the correct answer being “inappropriate” (Table 4). No associations exist between answering questions correctly and belonging to any of the majors or being in a pre-clinical or clinical year (Table 5). Chi-Square test was not performed on Nursing and Nutrition majors since in the former, there exists no pre-clinical phase (i.e., nursing students interact with patients from the very first year of their major), and in the latter, students do not interact with patients throughout the entirety of their undergraduate years.

**Table 5:** Percentage of students with one or no wrong answer to questions in Table 4 as per clinical phase and major

		Percentage of students who have no more than one wrong* answer to specific questions^†^ in Table 4			
Major	N	Percentage of students in a major (N)	Percentage of pre-clinical students (N)	Percentage of clinical students (N)	*p* Value
Medicine	123	47.2% (58)	52.9% (37)	39.6% (21)	0.145
Pharmacy	110	51.8% (57)	48.0% (12)	52.9% (45)	0.664
Dentistry	125	39.2% (49)	38.6% (17)	39.5% (32)	0.924
Nursing	28	42.9% (12)	N/A‡	42.9% (12)	N/A
Nutrition	30	46.7% (14)	46.7% (14)	N/A‡	N/A‡
Physiotherapy	39	56.4% (22)	28.6% (2)	62.5% (20)	0.101
Medical lab	28	50.0% (14)	53.3% (8)	46.2% (6)	0.705
Total	483	46.8% (226)	47.1% (90)	46.6% (136)	0.907

*Answers deemed inappropriate by international guidelines

^†^Questions 10 through 17 of Table 4

^‡^N/A = Not applicable

## Discussion

This study assessed the awareness of healthcare students at one institution regarding the professional use of social media. To date and to our knowledge, no study has compared the differences in the attitudes and practices of students belonging to various healthcare majors. All the participants used at least one social media platform, with Instagram and Facebook being the most utilized. Furthermore, we found that the average time spent on said platforms was no less than an hour per day, similar to an Australian study performed on doctors [10].

Most of the students in this study did not receive any form of structured education on the use of social media professionally during their university studies. This is compliant with the findings of a study from Saudi Arabia where most students did not receive classes related to social media professionalism [32]. We detected no significant differences in the level of awareness on social media professionalism between pre-clinical and clinical students of all faculties and majors (*p* value = 0.907). Contrastingly, at Oxford University, when students, regardless of their year of study, received education on professional social media guidelines, their awareness remarkably improved and their compliance with the guidelines enhanced [21]. This finding is also supported by numerous studies [5, 8, 10, 21, 22, 33–38] which emphasized the importance of structured education and the implementation of guidelines on the professional usage of social media among healthcare students and professionals. In the Arab and Middle Eastern region, studies on healthcare students also supported the need to develop clear guidelines delineating the safe and professional use of social media by future healthcare providers [32, 39–41].

The majority of the participants knew the current privacy settings of their accounts on social media, and the importance of this knowledge is supported by multiple international guidelines [27, 28, 42–47]. The results of this study showed that patients were more likely to send a friend request to a healthcare professional, but the opposite seldom occurred. This is reinforced by the findings of another study that showed that online patient-doctor interactions are more often than not started by the patients because healthcare workers do not feel at ease initiating interactions with patients through online platforms where privacy and security issues are far from ideal [48].

More than half of the participants considered posting pictures of themselves in bathing suits or while smoking or drinking alcohol as inappropriate, which is consistent with students’ perceptions from different fields [22, 35, 38], and professional international guidelines [27–29, 42, 43, 45–47, 49–51]. It should be noted that almost all professional international guidelines adopted in this study do not specify whether controversial online conduct is considered unprofessional on personal accounts, professional accounts, or both. This indistinction causes healthcare professionals and students to confuse personal with professional boundaries online, leading to inappropriate conduct and conflicts ending sometimes in dismissal from work or institution. A study on pharmacists recruited from numerous countries found that most of them did not adopt separate accounts for professional and personal matters on social media, with resultant negative outcomes on the professional career of the individuals [52]. Another problem faces healthcare professionals; even with separate personal and professional accounts, absolute privacy and control over the personal account do not exist as any content posted online can remain available in perpetuity and be misused or inappropriately interpreted by colleagues and patients [3, 42, 53].

Female students, regardless of their major, were more likely to adopt a professional attitude on social media. Kenny et al. [22] found similar results among female dental students. In our study, students from different majors had variable answers to three out of eight questions related to online professionalism, with no major showing absolutely better knowledge over the others. In our review of the current literature, we could not find any study tackling the differences in the online professional conduct between the students of different majors. However, we assume that students from all majors have comparable knowledge of social media professionalism and recommend creating and implementing standardized guidelines for students in the healthcare field. While many guidelines emphasized the similarity between the professional standards on online platforms and face-to-face interactions [27–29, 45, 46, 51, 54], the majority of the participants in this study regarded the professional standards as being different between the real and virtual worlds. This is perhaps due to the widespread misguidance of healthcare students in numerous countries on the professional use of social media and the hasty transcendence of professionalism from face-to-face consultations to online interactions [3, 12]. However, this blurring of the lines creates pitfalls in approaching professionalism on social media, especially since the usage of these platforms is high among healthcare professionals [55], and a wrong understanding of the value of such standards online can render professionals unaware of the ethical and legal obligations that they have towards their patients [10].

Except for a few, most students in this study were against mentioning any information online that may lead to a breach of provider-patient confidentiality. This indicates that the students, across different fields, were aware of the importance of maintaining patients’ privacy online, which is threatened on social media platforms [11]. More than half of the participants considered discussing political views online as unprofessional behavior. Although political affairs are frequently brought up on social media, guidelines have not clearly stated their position on this particular issue. Nevertheless, a 2016 study [56] revealed that patients with attitudes that disregarded the physician’s political views were treated differently which creates an undesirable discrepancy in patient care.

Despite opposing the idea of initiating an encounter with a patient online, around two-thirds of the participants affirmed that they would provide, in future practice, medical advice to a patient via social media. This answer contrasted the findings of two studies, one on medical students and physicians, and the other on pharmacists, which were both against interacting and giving medical advice to patients online [30, 48]. These matters, despite their importance, were either not discussed in some international guidelines or not clearly stated as right or wrong in others. The cloudiness surrounding these concerns creates an ambiguity worldwide among healthcare professionals and unpreparedness to face such popular requests on social media.

### Strengths and limitations

This study is the first of its kind in Lebanon and the first to be performed on healthcare students of different majors. However, it is not devoid of limitations. We conducted our research in only one university and the sampling technique was performed by convenience. Yet, we expect this institution to represent students from all the Lebanese population, in addition to reflecting the status of any healthcare student around the world who did receive any form of training or education regarding professionalism on social media. Additionally, we acknowledge that the response rate (27.54%) is potentially considered low for direct questionnaires. This occurred because of the settings of distributing the questionnaires: distribution occurred at the rest time between classes. Since certain questions required recalling past information, there is a potential for recall bias. Further, in some questions in the attitudes section, we were not able to compare the responses of the pre-clinical versus clinical students in the nutrition and nursing majors, because in the former, the clinical phase starts after graduation, and in the latter, the clinical phase begins in the first year of university studies. Lastly, as the field of social media is constantly evolving, it is practical to denote that the findings of this study reflected the situation at the time and place in which it was held.

## Conclusion

This study aimed to assess the awareness of healthcare students concerning social medical professionalism. In light of the study results, a pressing need emerges to accentuate the importance of creating and fostering a set of professional guidelines on the usage of social media in Lebanon and other countries that have yet to establish their guiding principles. These guidelines should be clear and accessible to all healthcare majors to help avoid pitfalls in the professional usage of social media and should underline the importance of legal and ethical matters that are often neglected on these platforms. Further research is needed to investigate efficient ways to teach the professional use of social media, especially that telehealth and electronic consultations are swiftly paving their way.

## Data Availability

The datasets used and/or analyzed during the current study are available from the corresponding author on reasonable request.

## Abbreviations

BAU: Beirut Arab University
BDA: British dietetic association
CSMLS: Canadian society for medical laboratory science
CSP: Chartered society of physiotherapy
IBM: International business machines
GDC: General dental council
GMC: General medical council
GPC: General pharmaceutical council
IRB: Institutional review board
NMC: Nursing and midwifery council
RPS: Royal pharmaceutical society
SPSS: Statistical package for the social sciences
